# Development of a psychometrically valid and reliable measure of primary care providers’ willingness to engage with value based payments and innovations in care coordination

**DOI:** 10.1186/s12913-024-11983-0

**Published:** 2024-12-18

**Authors:** Adam Atherly, Eline van den Broek-Altenburg, Alicia Jacobs, Josiah Mueller, Carrie Wulfman, Constance van Eeghen

**Affiliations:** 1 Department of Health Administration, College of Health Professions, Virginia Commonwealth University, 900 E. Leigh St, Richmond, VA 23219 USA; 2 Department of Radiology, Larner College of Medicine, University of Vermont, Burlington, VT USA; 3 Department of Family Medicine, Larner College of Medicine, University of Vermont, Burlington, VT USA; 4 Value-Based Care, OneCare Vermont, Burlington, VT USA; 5 Larner College of Medicine, University of Vermont, Burlington, VT USA

**Keywords:** Value based care, Accountable care organizations, New instrument, Factor analysis, Provider engagement, Primary care

## Abstract

**Background:**

Most approaches to healthcare reform envision an enhanced role for primary care providers, supported by innovative payment methodology and improved resources. However, there are currently no instruments to measure providers’ ability and willingness to work with existing tools provided by payers, such as Accountable Care Organizations (ACO). In this study, we develop and psychometrically test a new instrument to measure provider engagement with ACOs.

**Methodology/ approach:**

The instrument was developed based on a self-efficacy theory of the adoption of innovations. We hypothesized two underlying constructs: *Ease of Use* (“Ease”) and *Perceived Usefulness* (“Usefulness”). Constructs were tested using confirmatory factor analysis. Reliability was assessed with Cronbach's Alpha and convergent and divergent validity. Survey subjects were Primary Care Providers engaged with an ACO.

**Results:**

Eigenvalue and scree plots indicated the hypothesized two factor model was appropriate. Four questions failed to load onto a single factor – three from Ease and one from Usefulness. Both scales have outstanding reliability, with an Alpha of 0.951 for *Usefulness* and 0.831 for *Ease*. For validity, the results are consistent with our prior hypotheses for convergent and divergent validity.

**Conclusions:**

The new instrument is a valid and reliable measure of providers’ ability to work with and gain value from ACO participation.

**Practice implications:**

The success of any health care reform will be highly dependent on primary care providers’ willingness and ability to engage with payers. This instrument provides a new tool to measure the value and difficulty of that engagement by primary care providers.

**Supplementary Information:**

The online version contains supplementary material available at 10.1186/s12913-024-11983-0.

## Introduction

Many approaches to healthcare reform in the United States envision an enhanced role for primary care providers [1]. Research has shown that robust primary care can lead to improved health outcomes, increased health equity and lower total healthcare spending [2–7], with particular cost effectiveness in older frail adults [8]. Population health efforts, specifically, depend on high quality accessible primary care [9, 10].

Yet, despite the importance of primary care, primary care practices have been shrinking for many years, with 65 million Americans now living in primary care shortage areas [11]. Primary care practices are struggling both financially and in delivering care [12]. Declines in the supply of primary care providers [11] and quality of the provider-patient relationship [13] are widespread, and are coupled with increases in primary care provider burnout [14].

This creates a conundrum: primary care is the lynchpin for many ideas for healthcare reform, particularly population health-based reforms, yet primary care practices are themselves struggling. Indeed, one of the areas where primary care practices have difficulty is in innovation [15] – precisely what policymakers desire from primary care practices.

There are many ideas for how to improve and strengthen primary care so that it can assume its envisioned role in a reformed healthcare system. The Agency for Healthcare Research and Quality (AHRQ) lists a series of options, ranging from improved care coordination to integration of behavioral health [16]. Other ideas for improving primary care include everything from new data "dashboards" to improved care coordination to integrated mental health services [17]. These ideas for improving primary care are beginning to lead to concrete policy proposals. For example, the American Academy of Family Physicians is engaged in a three-year project to develop value-based payments for primary care [18] and Medicare recently announced a shift toward increasing primary care payments [19].

All of these reform ideas envision that primary care providers can change their behavior in response to different incentives, data or resources. Yet this presumed model of behavioral change is potentially misaligned with the reality of day-to-day provider practice. Primary care practices already face a shortage of primary care providers, which leads to longer wait times for appointments [11]. Additionally, the fee-for-service (FFS) payment model often discourages comprehensive and preventive care, prioritizing volume over patient outcomes [19]. Furthermore, the increasing prevalence of chronic diseases and an aging population demands more time and resources for patient management. The integration of electronic health records (EHRs) and the burden of administrative tasks (EHR and other non-reimbursed care) divert providers from patient-centered care.

Addressing these challenges requires significant policy reforms, innovative care models, and increased investment in primary care. Yet it also requires significant engagement from primary care providers. A new payment model or dashboard will only be effective if providers are aware of the change and respond to the new information or incentives through changes in care delivery.

One critical measure of the likelihood of success in healthcare reform focused on primary care will be the extent to which primary care providers accept, understand and adopt care enhancements. There has been considerable research on challenges associated with primary care practices adapting to new technology and care enhancements. While some practices were able to quickly incorporate new technologies and enhance efficiencies [20], there is provider level variation in adoption even within successful adopting agencies [21]. Challenges to successful adoption ranged from hardware integration to workflow issues [21]. Effective adoption requires a clear understanding of the target users, their specific data needs and the development of structures to facilitate daily use [22]. The rapid transition to telemedicine during the Covid-19 pandemic highlighted both opportunities and challenges for primary care providers, including the need for adequate training to effectively navigate remote consultations, guidelines and attention to technological barriers [23–25].

A similar challenge for providers is presented by Accountable Care Organizations (ACOs). ACOs aim to improve patients’ healthcare experience while also containing healthcare costs, but are highly dependent on their ability to overcome fragmentation of care by linking providers across the care continuum, with primary care providers at the center [26]. Primary care providers (PCPs) see many potential benefitsfrom ACOs – including care coordination, data analytics and improved communication with other providers – but are concerned about the potential administrative, time and other challenges associated with participation [27].

To date, there are no published measures of primary care provider engagement with healthcare reforms like ACOs. This paper fills that gap by developing a psychometrically reliable and valid measure of primary care providers’ engagement with care flexibility associated with a new payment methodology and accompanying technology to support that flexibility. In this paper, we present a new instrument to measure the willingness of primary care providers to adopt novel approaches to care when provided new technology and payment flexibility. The new instrument measures provider’s perception of the ease and value of engagement with an ACO.

## Theory

Most primary care reforms offer some combination of flexible payment and improved data for providers to manage patients (e.g., “dashboards”). Our theory is that primary care providers’ willingness to accept the new flexibility and information will be similar to prior work measuring “acceptance of new technology”. Early work studied acceptance of new technology in the context of (then new) email and computer based graphic systems [28], which in turn was based on earlier research on the impact of perceived usefulness on system utilization [29, 30]. In that framework, it was assumed that new systems that failed to help workers perform their jobs were unlikely to be successful. As discussed in Davis [28], the theoretical framework could be derived from a number of different theoretical paradigms, including self-efficacy theory, the cost–benefit paradigm and adoptions of innovations.

In our context, this suggests that primary care providers will adopt new technology – care flexibility through alternative payment methods or dashboards, for example – if they perceive that the changes are potentially useful, and the benefits exceed the costs. Benefits and costs can both be thought of in terms of money, time, practice resources or patient outcomes.

Each of the theoretical paradigms is consistent with the idea that the acceptance of new technology can be measured in two different domains: *Ease of Use* (Ease) and *Perceived Usefulness* (Usefulness). Ease is defined as *“the degree to which a person believes that using a particular system would be free of effort”,* while Usefulness is defined as *“the degree to which a person believes that using a particular system would enhance his or her job performance”*. In prior work studying information technology adoption, these two domains were found to be predictive of willingness to adopt a new technology.

## Methods

Our goal was to develop a measure of provider engagement with an accountable care organization and the tools provided by that organization. To develop the measure, we used a multi-step, multi-phase measurement development methodology. We selected the measure developed by Davis (1989) on ease of technological adoption as our framework [28]. This selection was guided by our conceptual framework based on the hypothesis that willingness to adopt new primary care practice patterns in response to new technologies and new payment methodologies would map to two domains similarly to technology adoption. Our goal was to extend these ideas to adoption of new approaches to primary care.

## Setting

Our study is based in Vermont. The state of Vermont received a state-wide All-Payer Model (APM) waiver from Center for Medicare and Medicaid Innovation (CMMI) in 2017 which allows for a global payment that includes all-payers, including Medicare, Medicaid, and most commercial payers (by statute) [31, 32]. The global all-payer reimbursement mechanism is exclusive to Vermont and nationally unique in that all contracts pay a prospective actuarially determined “all-inclusive population-based payment” monthly for all anticipated inpatient, hospital outpatient and professional services for the attributed beneficiaries. The waiver is part of an ambitious effort to fundamentally alter the misalignment of payment incentives across all payers and create an environment where care providers can shift their focus from revenue generation to population health.

Participation in the APM by primary care practices is voluntary. Members are attributed to the APM only if their primary care provider has voluntarily entered into a participation contract. Primary care practices have a series of decisions regarding whether and how to participate. Practices can simply opt out. Alternatively, practices can participate for some contracts (e.g., Medicaid) but not others (e.g., Medicare). Alternatively, practices can fully participate but retain FFS payment. Finally, practices can fully participate and select an alternative payment mechanism, such as full capitation for attributed lives.

Participation in the APM provides several advantages. The Vermont APM telemedicine rules mimic the Medicare ACO Next Generation model in that pre-COVID-19, audio/video telemedicine visits with at-home patients were allowed for attributed lives only. The APM also provides practices with funds for care coordination. Participating practices receive per member per month funds for care coordination, with wide latitude on how the funds can be spent. Independent practices that fully engage with the ACO have the option to move away from FFS payment. Practices also receive population health management payments of “per attributed beneficiary per month” and data on vulnerable patients, including an app designed specifically to aid in managing patients remotely during COVID-19.

Early studies of the APM have found generally positive results. The five-year review of the program found that the APM reduced overall spending for the Medicare population and reduced both hospitalizations and readmissions [33]. Stakeholder analysis suggested that the APM also improved system level collaboration and was able to extend and expand existing efforts at health system transformation.

The Vermont reform, similar to any ACO reform, relies on primary care providers to alter care delivery in response to the new incentives and resources. But whether providers act on the new incentives or not is unknown. To date, there are no existing instruments to measure providers’ ability and willingness to work with tools and opportunities provided by the APM.

### Approach

We adapted Davis’ initial measure to reflect the healthcare domain of provider engagement. The developed measures were then reviewed by six subject matter experts from the payer, including perspectives of practice network operations, legal and managed care contracting, payment reform, policy development, population health, and primary care. Based on feedback from the reviewers, the measure was improved and then pretested with four primary care provider respondents.

After reviewing the pilot test results and qualitatively interviewing the pilot testers, the survey instrument was finalized. Two questions were removed from the survey: *Participating with [ACO] enhances my effectiveness on the job* from Usefulness and *Overall, I find [ACO] easy to work with* from Ease. In both cases, the pilot testers found the questions duplicative with other measures. The final survey instrument is available in the supplementary files, with the name of the ACO redacted.

The tested instrument included 13 questions measuring Perceived Usefulness: “*the degree to which a person believes that using a particular system would enhance his or her job performance*”, and seven questions measuring Ease of Use: “*the degree to which a person believes that using a particular system would be free of effort*” (Table 1). All questions were framed on a 1–5 Likert scale from “strongly agree” to “strongly disagree”. Approximately half the questions used reverse coding, which were recoded during analysis so that a higher score indicates higher engagement. In creating the overall score, items were summed after the recoding with all items equally weighted.

**Table 1:** Initial items for the survey

**Usefulness of ACO:**
1. My job would be more difficult to perform without [ACO]
2. [ACO] gives me greater control over my work
3. Working with [ACO] improves the quality of care I deliver
4. [ACO] data services address my care delivery needs
5. Working with [ACO] saves me time
6. [ACO] enables me to accomplish tasks more quickly
7. [ACO] supports critical aspects of my job
8. Participating with [ACO] allows me to accomplish more work than would otherwise be possible
9. Participating with [ACO] reduces the time I spend on unproductive activities
10. Participating with [ACO] enhances my effectiveness on the job
11. Participating with [ACO] increases my productivity
12. Participating with [ACO] makes it easier to deliver care
13. Overall, I find [ACO] useful for care delivery
**Perceived Ease of Use**
1. I am often confused about the services offered by [ACO]
2. Interacting with [ACO] is often frustrating
3. Interacting with [ACO] requires a lot of effort
4. [ACO] is rigid and inflexible to interact with
5. I find it easy to get [ACO] to do what I want it to do
6. I find it cumbersome to work with [ACO]
7. [ACO] often behaves in unexpected ways

We also asked our respondents a number of questions about their understanding of and exposure to the APM, the respondent’s perception of the proportion of patients in the APM, and information about themselves (Physician, Advanced Practice Nurse, Other) and practice type (independent group practice, independent individual practice, hospital based and FQHC).

#### Survey process

The survey items were entered into the REDCap survey system after pretesting [34, 35]. The APM ACO sent a preparatory email to key contacts at each primary care practice to let them plan for distribution of the survey to their primary care providers and inform them that the survey was sanctioned by the ACO. The REDCap survey link was delivered to these same contacts a week later. Respondents were provided with an overview of the purpose of the survey and were allowed to opt out of participation. All responses were anonymous. According to the policy defining activities which constitute research at the University of Vermont, this work met criteria for operational improvement activities not requiring Institutional Review Board review. Surveys were initially sent out on August 1, 2022. Four different reminders were sent out to improve the response rate. The survey was formally closed on September 30, 2022.

### Scale development, reliability and validity

The underlying constructs were identified using a confirmatory factor analysis. Factor analysis is a multivariate technique that identifies common response patterns among a set of items. “Confirmatory” indicates that there were pre-established hypotheses about how the items were intended to load. Table 1 indicates our prior hypotheses about the expected loadings and was developed prior to the survey deployment. We used oblique rotations to allow correlations among the underlying constructs.

Reliability was established via Cronbach’s Alpha. In general, an Alpha above 0.8 is considered acceptable. A different Alpha was calculated for each scale. Validity was established by testing statistical relationships that were hypothesized prior to the analysis. We hypothesized that *Usefulness* would be positively associated with the perceived proportion of the patient population in the ACO, being in the ACO by Choice and being rewarded for good outcomes, but *Ease* would not. The logic of our hypothesis is that (for example) the data would be more valuable and thus useful if a practice was being rewarded for particular outcomes measured by those data, but that being rewarded for care would not necessarily make the data easier to work with. We also hypothesized that the percentage of patients in the ACO should have the highest correlation with *Ease*, with the logic that experience would help providers learn how to work with the APM ACO more easily – although we did not hypothesize any directionality for that correlation.

## Results

We received a total of 115 responses, out of a maximum possible 944 primary care providers in the network (total number of actual recipients to receive survey invitations is unknown), yielding a conservatively estimated response rate of 12%. Of these, 114 agreed to participate, but only 71 completed the survey. Physicians represented 80% of responses and 20% identified as Advance Practice Nurses (one response identified as “other”). The sample size was sufficient for the factor analysis and to test the measure for reliability and validity.

### Factor analysis results

**Fig. 1:**
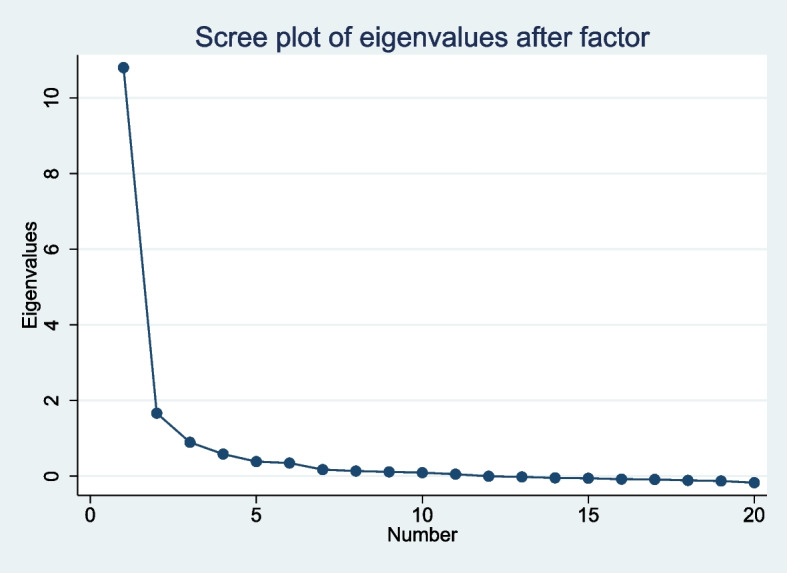
Scree plot of eigenvalue after factor analysis showing number of factors

The factor analysis includes two different statistics: the Factor Loading (for each factor) and the Uniqueness. Effective Items should load on a single factor, showing that they distinguish between the two underlying constructs. We tested the number of factors and rotations to find the best fit for the data. The commonly used scree plot of eigenvalues was consistent with two underlying factors per the prior expectations (Fig. 1). The factor loadings were also consistent with our prior expectations.

The results (Table 2) suggest that one item should be removed from the *Ease* scale (out of nine):

- I am often confused about the services offered by [ACO]

**Table 2:** Items selected for analysis with factor loadings and uniqueness items

	Factor 1	Factor 2	Uniqueness
My job would be more difficult to perform without [ACO]	0.7052	0.2242	0.4525
[ACO] gives me greater control over my work	0.7119	0.1712	0.4638
Working with [ACO] improves the quality of care I deliver	0.875	0.083	0.2275
[ACO] data services address my care delivery needs	0.6711	−0.0042	0.5496
Working with [ACO] saves me time	0.8068	0.0932	0.3404
[ACO] enables me to accomplish tasks more quickly	0.8343	0.0934	0.2952
[ACO] supports critical aspects of my job	0.764	0.2194	0.3682
Participating with [ACO] allows me to accomplish more work than would otherwise be possible	0.8667	0.1682	0.2205
Participating with [ACO] reduces the time I spend on unproductive activities	0.8479	0.1174	0.2673
Participating with [ACO] enhances my effectiveness on the job	0.8586	0.2575	0.1966
Participating with [ACO] increases my productivity	0.9018	0.1585	0.1617
Participating with [ACO] makes it easier to deliver care	0.9075	0.1338	0.1585
Overall, I find [ACO] useful for care delivery	0.8848	0.166	0.1895
Interacting with [ACO] requires a lot of effort	−0.3916	0.6213	0.4606
I am often confused about the services offered by [ACO]	−0.388	0.21	0.8053
[ACO] is rigid and inflexible to interact with	−0.6152	0.3755	0.4805
I find it cumbersome to work with [ACO]	−0.5299	0.4635	0.5044
Interacting with [ACO] is often frustrating	−0.6299	0.3707	0.4658
[ACO] often behaves in unexpected ways	−0.3556	0.4691	0.6535
I find it easy to get [ACO] to do what I want it to do	−0.7554	0.4586	0.2191

This left thirteen items retained on the Usefulness scale and six on the Ease scale for a total of 19 items.

### Reliability and validity

Reliability was tested using Cronbach’s Alpha. Both scales have outstanding reliability, with an Alpha of 0.951 for *Usefulness* and 0.831 for *Ease*.

For validity, correlations were calculated between the scales (summed up at the individual level) and the three test variables: Patient Percentage, ACO Membership by Choice and ACO rewards for good outcomes. The results are shown in Table 3. For *Usefulness*, the highest correlations were for *ACO Rewards for Good Outcomes*, followed by *ACO Membership by Choice* and *Patient Percentage.* For the *Ease* scale, the opposite pattern is observed. These results are consistent with our a priori hypotheses.

**Table 3:** Validity correlations testing validity of instruments

	**Usefulness**	**Ease**
Patient Percentage	0.16	−0.09
ACO Membership by Choice	0.268	−0.216
ACO Rewards for Good Outcomes	0.561	−0.557

The *Ease of Use* scale includes six items on a 1–5 scale, with higher numbers indicating an easier time working with the ACO; the *Perceived Usefulness* included thirteen items on a 1–5 scale, with higher numbers indicating a more productive time working with the ACO.

## Discussion

In this study, we developed a measure of provider engagement with payers and tools with an accountable care organization. We hypothesized that provider engagement with payers and tools would be characterized by two different latent constructs, which we found to be the case. The developed scale has 19 questions across two different domains: 1) Perceived Usefulness: “*the degree to which a person believes that using a particular system would enhance his or her job performance*” with 13 Questions, range 13–65 and 2) Ease of Use: “*the degree to which a person believes that using a particular system would be free of effort*”, with six Questions, range 6–30. The scale has demonstrated validity and outstanding reliability.

The developed scale is a tool that can be used to assess providers’ acceptance of new care delivery and technology options and also assess improvements in acceptance and use over time. The prior literature discussed in the introduction highlighted the variability of implementation success of new care delivery and technology innovations both across and within organizations. Reported challenges included navigating new technologies, adapting workflows and receiving appropriate training.

This tool can help measure providers’ views on these challenges and new care innovations and technology, giving early indications about the likelihood of success and identifying areas to target training and guidelines. Repeated measures over time can evaluate the success of the implementation and provider acceptance of the changes. Having providers accept and be adept with the new innovations and technologies does not guarantee success for these changes, but success is unlikely if providers are unwilling or unable to effectively adapt to the new environment.

This study does have several limitations. The analysis was limited to primary care clinicians, so the validity of the measure for other provider types is unknown. As the mechanics of survey distribution in the ACO network was dependent on key contacts at the primary care practices for distribution to primary are providers, it is not possible to verify who received the survey. We selected this approach because we believed it would provide the highest response rate. We also used language from the instrument tested by Davis and decided to adhere closely to this work and to the underlying theoretical construct; alternative wording could be considered for some questions. Finally, our analysis has a relatively low sample size for a factor analysis. Additional research confirming the results of this analysis would be useful.

Given the centrality of PCP engagement with care innovation for healthcare reform to be successful, particularly for ACOs, it is important to measure provider willingness to engage with payers and tools. This instrument can be used by ACOs or managed care organizations to measure provider’s perceptions of the value and ease of use of different tools provided to aid in care delivery. These can then be used to develop metrics that can be tracked over time to measure the success of new payment and care delivery processes.

## Practice implications

Healthcare reform requires active participation by frontline providers. Prior research suggests that changing health delivery and practice care patterns is challenging. Successful reforms will need provider engagement. Our measure provides a methodology to measure primary care provider engagement with payers and can play a role in measuring the success of health reform efforts.

## Supplementary Information

Supplementary Material 1.

## Data Availability

The datasets used and/or analyzed during the current study are available from the corresponding author on reasonable request.
