# Supplementary material for: Development of a psychometrically valid and reliable measure of primary care providers’ willingness to engage with value based payments and innovations in care coordination
Source: BMC Health Serv Res. 2024 Dec 18;24:1562. doi: 10.1186/s12913-024-11983-0 (PMC11653968; doi:10.1186/s12913-024-11983-0)
Supplement: Supplementary file 1 — Supplementary Material 1. [file 12913_2024_11983_MOESM1_ESM.pdf]

Redacted Provider Survey

Introduction

XXXXXX has asked the University of Vermont to evaluate its impact on primary care delivery in Vermont. You were selected to take part in this evaluation because of your participation in XXXXXX.

Project Procedures

If you take part in the project, you will be asked to answer a short series of questions about how XXXXXX impacts your delivery of primary care.

Time

The survey will take approximately 5 minutes of your time.

Risks and Benefits

There are no risks associated with this project. You have been contacted by email, but this identifying information will only be used to provide you with the survey link and will be deleted at the end of the project. There is the risk of a breach of confidentiality that the project team will take steps to minimize, as noted in the confidentiality section below.

There will be no costs to you for participation in this project other than your time. You will not be paid to take part in this project. You will not receive any direct benefits from participating in this survey other than service improvements XXXXXX intends to make as guided by survey responses.

.

Confidentiality

Identifying information from the survey will only be used by the UVM project team. Your permission to use your responses will not expire unless you tell us you want to cancel it. The project will continue to use the survey information already collected for the project before you cancelled your permission and you cannot get back information that was already shared with others. No identifiable information will be shared outside the project team.

Voluntary Participation/Withdrawal

Taking part in this project is voluntary. You are free to not answer any questions and can withdraw at any time. If you choose to stop taking part in this project, you may cancel permission for the use of your survey responses at any time. You must let the project team know that you are cancelling your permission. A member of the project team will assist you in making your decision effective.

Questions

If you have any questions about this project now or in the future, you may contact the project director, Dr. Connie van Eeghen via the email address Constance.Van-Eeghen@med.uvm.edu. If you have questions or concerns about your rights as a project participant, you may contact the Director of the Research Protections Office at (802) 656-5040.

If you decide to participate in the project, we ask you to take all questions seriously and honestly. Data are collected for the purposes of evaluation and improvement and to inform public health policy. Keep in mind that your participation is voluntary, and you can at any time decide to stop answering the survey.

By clicking on "Yes- I would like to participate in the survey" you give your consent to take part in the project and the survey will be launched.

◯ Yes - I would like to participate in the survey
◯ No

Please indicate the extent to which you agree or disagree with the following statements

	Strongly Agree	Agree	Neither Agree nor Disagree	Disagree	Strongly Disagree
My job would be more difficult to perform without XXXXX	○	○	○	○	○
XXXXXX gives me greater control over my work.	○	○	○	○	○
Working with XXXXXX improves the quality of care I deliver.	○	○	○	○	○
XXXXXX data services address my care delivery needs.	○	○	○	○	○
Working with XXXXXXX saves me time.	○	○	○	○	○
XXXXXX enables me to accomplish tasks more quickly.	○	○	○	○	○
XXXXXX supports critical aspects of my job.	○	○	○	○	○
Participating with XXXXXX allows me to accomplish more work than would otherwise be possible.	○	○	○	○	○
Participating with XXXXXX reduces the time I spend on unproductive activities.	○	○	○	○	○
Participating with XXXXXX enhances my effectiveness on the job.	○	○	○	○	○
Participating with XXXXXX increases my productivity.	○	○	○	○	○
Participating with XXXXXX makes it easier to deliver care.	○	○	○	○	○
Overall, I find XXXXXX useful for care delivery.	○	○	○	○	○

Please indicate the extent to which you agree or disagree with the following statements:

	Strongly Agree	Agree	Neither Agree nor Disagree	Disagree	Strongly Disagree
I find it easy to get XXXXXX to do what I want it to do.	○	○	○	○	○
Interacting with XXXXXX requires a lot of effort.	○	○	○	○	○
I am rarely confused about the services offered by XXXXXX.	○	○	○	○	○
XXXXXX is rigid and inflexible to interact with.	○	○	○	○	○
XXXXXX provides helpful guidance in performing tasks.	○	○	○	○	○
I find it cumbersome to work withX XXXXXX.	○	○	○	○	○
Interacting with XXXXXX is rarely frustrating.	○	○	○	○	○
XXXXXX often behaves in unexpected ways.	○	○	○	○	○
Overall, I find XXXXXX easy to work with.	○	○	○	○	○

Please indicate the extent to which you agree or disagree with the following statements:

	Strongly Agree	Agree	Neither Agree nor Disagree	Disagree	Strongly Disagree
I know what XXXXXX does	◯	◯	◯	◯	◯
I am a member of XXXXXX by choice	◯	◯	◯	◯	◯
XXXXXX rewards me for good outcomes	◯	◯	◯	◯	◯

Which of the following best describes the type of practice where you work:

- ◯ Independent Group Practice
- ◯ Independent Individual Practice
- ◯ Hospital based Practice
- ◯ FQHC
- ◯ Other

How would you describe your practice type? ______________________________

Which of the following best describes your professional background:

- ◯ Physician
- ◯ Advanced Practice Provider
- ◯ Other

How would you describe your professional background? ______________________________

What percentage of patients you provide care for do you think are attributed to XXXXXX? ______________________________

What is the zip code of the primary practice where you work? ______________________________

Practice name: ______________________________

In which Health Service Area (HSA) are you located? ______________________________
